# Efficient Numerical Simulation of Biochemotaxis Phenomena in Fluid Environments

**DOI:** 10.3390/e25081224

**Published:** 2023-08-17

**Authors:** Xingying Zhou, Guoqing Bian, Yan Wang, Xufeng Xiao

**Affiliations:** 1 School of Future Technology, Xinjiang University, Urumqi 830046, China; zxy128280@163.com (X.Z.); guoqing-bian@foxmail.com (G.B.); 2 College of Mathematics and System Sciences, Xinjiang University, Urumqi 830046, China; wangyanmath@yeah.net

**Keywords:** biochemotaxis, dimension splitting method, finite difference method, 92C10, 65M06, 65Y05

## Abstract

A novel dimension splitting method is proposed for the efficient numerical simulation of a biochemotaxis model, which is a coupled system of chemotaxis–fluid equations and incompressible Navier–Stokes equations. A second-order pressure correction method is employed to decouple the velocity and pressure for the Navier–Stokes equations. Then, the alternating direction implicit scheme is used to solve the velocity equation, and the operator with dimension splitting effect is used instead of the traditional elliptic operator to solve the pressure equation. For the chemotactic equation, the operator splitting method and extrapolation technique are used to solve oxygen and cell density to achieve second-order time accuracy. The proposed dimension splitting method splits the two-dimensional problem into a one-dimensional problem by splitting the spatial derivative, which reduces the computation and storage costs. Finally, through interesting experiments, we show the evolution of the cell plume shape during the descent process. The effect of changing specific parameters on the velocity and plume shape during the descent process is also studied.

## 1. Introduction

Biochemotaxis models are used to describe oxidation patterns of some cells in nature. These cells tend to inhabit viscous fluids. The cells and chemical attractants are moved about with the fluid. The gravity produced by the cell aggregation then has an impact on the fluid’s flow. Therefore, the biochemotaxis equation is usually coupled to the incompressible Navier–Stokes (NS) equation that governs fluid motion. The aim of this study is to develop an effective second-order dimension splitting scheme for the chemotaxis-Navier–Stokes equations proposed by [1,2]. This scheme is designed to achieve the accurate and fast numerical simulation of cell biochemotaxis. The chemotaxis-Navier–Stokes model includes a reaction–diffusion equation that involves changes in oxygen concentration, a convection–diffusion equation that describes how cells travel along an oxygen concentration gradient, and an incompressible Navier–Stokes equation that governs the motility of the fluid.

We now introduce the dimensionless chemotaxis-Navier–Stokes equation and define the dimensionless parameters. The equation is defined in terms of non-negative parameters, namely α, β, γ, and ζ, as well as the Schmidt number $S_{c}$. We briefly outline these parameters and their meaning in the equation:

(1) $$\begin{array}{c} \alpha =\frac{\chi_{q}c_{air}}{\alpha_{n}},\;\beta =\frac{q_{r}L^{2}}{c_{air}\alpha_{n}},\;\gamma =\frac{V_{b}q_{r}g\left(\rho_{b}-\rho \right)L^{3}}{\eta \alpha_{n}},\;\zeta =\frac{\alpha_{c}}{\alpha_{n}},\;S_{c}=\frac{\eta }{\alpha_{n}\rho }, \end{array}$$

where *L* and $q_{r}$ are characteristic length and characteristic cell density, respectively. $c_{air}$ is the air’s oxygen concentration. $V_{b}$ is the volume of the cell, $g=9.8\;m/s^{2}$ is the gravitational acceleration, and $\rho_{b}$ is the density of the cell [3]. In addition, the diffusion coefficients $\alpha_{n}$, $\alpha_{c}$ and the chemotactic sensitivity $\chi_{q}$ are non-negative. In a smooth, open, bounded, and square two-dimensional domain $\Omega \subset R^{2}$ and a time interval (0,T], the dimensionless chemotaxis-Navier–Stokes system is as follows:

(2) $$\left\{\begin{array}{cc} \frac{\partial q}{\partial t}+u\nabla q-∆q+\alpha \nabla \cdot \left(qr\left(c\right)\nabla c\right)=0, & \;\mathrm{in}\;\Omega \times (0,T],\\ \frac{\partial c}{\partial t}+u\nabla c-\zeta ∆c+\beta r\left(c\right)q=0, & \;\mathrm{in}\;\Omega \times (0,T],\\ \frac{\partial u}{\partial t}+\left(u\cdot \nabla \right)u-S_{c}∆u+S_{c}\nabla p=-f\left(q\right), & \;\mathrm{in}\;\Omega \times (0,T],\\ \nabla \cdot u=0, & \;\mathrm{in}\;\Omega , \end{array}\right.$$

where the variable $q=q\left(x\right)$ denotes the cell density with $x=\left(x_{1},x_{2}\right)$ and the oxygen concentration is indicated by the variable $c=c\left(x\right)$. The variables $u={\left(v_{1},v_{2}\right)}^{T}$, and $p=p\left(x\right)$ are defined as the velocity and the pressure of the incompressible NS equation with a density ρ and viscosity η, respectively. Moreover, $f\left(q\right)={\left(0,S_{c}\gamma q\right)}^{T}$ and: 

(3) $$r\left(c\right)=\frac{1}{2}\left(\tanh\left(\frac{c-0.3}{\epsilon }+1\right)\right),$$

where ϵ>0 is a small constant. Let ∂Ω be the boundary of computational domain Ω. The boundary ${\partial \Omega }_{top}$ represents the fluid–air interface. ${\partial \Omega }_{top}$ is the boundary at the bottom of computational domain Ω and ${\partial \Omega }_{lr}$ is the boundary at the left and right of computational domain Ω. On the ${\partial \Omega }_{top},$ there is no cell flux and the air’s oxygen concentration $c_{air}$ has saturated. In this paper, the numerical simulation is considered in a closed area with only the top contacting air, so the boundary conditions are set as:

(4) $$\left\{\begin{array}{c} \alpha r\left(c\right)q\frac{\partial c}{\partial x_{2}}-\frac{\partial q}{\partial x_{2}}=0,\;c=1,\;u=0,\;\;\mathrm{on}\;\partial \Omega_{top}\times \left(0,T\right],\\ \frac{\partial q}{\partial x_{2}}=\frac{\partial c}{\partial x_{2}}=0,\;u=0,\;\;\mathrm{on}\;\partial \Omega_{\mathrm{bot}\;}\times \left(0,T\right],\\ \frac{\partial q}{\partial x_{1}}=\frac{\partial c}{\partial x_{1}}=u=0,\;\;\mathrm{on}\;\partial \Omega_{lr}\times \left(0,T\right]. \end{array}\right.$$

In addition, the initial conditions are:

(5) $${\left.\;u\right|}_{t=0}=u_{0},{\left.\;q\right|}_{t=0}=q_{0},{\left.\;c\right|}_{t=0}=c_{0}.$$

As shown above, the chemotaxis-Navier–Stokes system is a non-linear, coupled system with multiple variables. To efficiently solve this system, it is important to decouple the equations. Additionally, a fine-mesh discretization is necessary for achieving high-resolution results in the two-dimensional case. However, it may result in high storage requirements and computational complexity. Thus, achieving high resolution while maintaining stability and computational efficiency remains an important research field.

In response to numerical instabilities and maintaining algorithmic accuracy, researchers have developed many effective numerical methods. In [4], a high-resolution hybrid finite-volume finite-difference method is developed for the chemotaxis–fluid system. The method takes two different approaches to study two different parts of the system (chemotaxis and fluid). In [3], based on the linear finite element method, Huang et al. applied the flux-corrected transport algorithm to the chemotactic equation to ensure high resolution and stability. In [5], the chemotactic stage uses a high-resolution method (from CLAWPACK) that captures steep gradients, whereas the diffusion step employs the stable TR-BDF2 methods. In [6], to achieve stability, a finite volume approach based on a second-order central upwind structure is used. Recently, Wang et al. established a new convergence-fluid diffusion domain (cf-DD) model, which is derived using a diffusion domain, and developed a second-order hybrid finite-volume finite-difference method to ensure correctness and high resolution [7]. The above works mainly consider the stability and high precision of numerical solutions. For two-dimensional problems, standard spatial discretization is performed directly in two-dimensional space, which has high computational complexity and storage requirements. In this paper, the dimension splitting method is used to solve the computational efficiency problem.

In response to the huge storage and computational effort required for two-dimensional discretization, the dimension splitting method is developed. The key is to split the operator according to the direction of the spatial derivative. An early dimension splitting method was the alternate direction implicit (ADI) method, which relied heavily on the finite difference method [8,9]. Operator splitting can be used in combination with most common numerical methods, so it is widely chosen as a dimension reduction method. For example, the operator splitting method is used for the convection Cahn–Hilliard equation [10], the Allen–Cahn-type equation [11], and the phase field crystal equation [12].

Our innovation is to establish a fast and low-cost numerical method for the chemotaxis-Navier–Stokes equation by using the dimension splitting method. First, a second-order pressure correction scheme with splitting effect is used to decouple the NS equation. The key is to use the operator $A=\left(I-\partial_{xx}\right)\left(I-\partial_{yy}\right)$ instead of the elliptic operator to correct the pressure [13], so that the decoupled two-dimensional pressure equation can be split into one-dimensional problem for computation. The ADI scheme is then used to solve the decoupled velocity field equation. For the chemotaxis equation, by dividing the second-order spatial partial derivatives, the sequential splitting method is employed to the dimensionality reduction, and then the time accuracy is improved to the second-order by an extrapolation method. The proposed method decomposes the two-dimensional equation into one-dimensional PDE subproblems. The computation of one-dimensional subproblems in the same direction is independent, so that parallel computation can be performed, which greatly reduces the computation and storage effort. For space discretization, the MAC finite difference scheme is considered [14,15].

The rest of the paper is organized as follows. In Section 2, we show the dimension splitting method for the coupled system of chemotaxis–fluid equations and incompressible Navier–Stokes equations. In addition, the marker-and-cell (MAC) finite difference scheme is introduced. In Section 3, we show some numerical experiments to demonstrate the efficacy of the suggested strategy. The concluding Section 4 of the article provides a comprehensive summary of the findings and discussions from the preceding sections, encapsulating the key takeaways and observations.

## 2. Numerical Schemes

In this section, we present the dimension splitting scheme of the chemotaxis-Navier–Stokes model and the second-order central difference scheme in the MAC grid configuration.

### The Dimension Splitting for the Chemotaxis-Navier–Stokes System

We give a second-order pressure-stabilization perturbation scheme for the NS equation in (6):

(6) $$\left\{\begin{array}{c} \frac{\partial u}{\partial t}+\left(u\cdot \nabla \right)u+S_{c}\nabla p-S_{c}∆u=-f\left(q\right),\\ \delta tA\psi +\nabla \cdot u=0,\\ \delta tp_{t}=\psi -\chi \eta \nabla \cdot u,\\ {\left.\frac{\partial \psi }{\partial n}\right|}_{\Omega }=0,\\ {\left.u\right|}_{\partial \Omega }=0, \end{array}\right.$$

where $A=\left(I-\partial_{xx}\right)\left(I-\partial_{yy}\right)$ and 0<χ≤1. The operator A replaces the Poisson operator in the majority of the literature, so the two-dimensional pressure equation is decomposed into one-dimensional subproblems to compute. In addition, this method not only has the advantage of high efficiency, but also has the same stability and convergence of Poisson projection (reference [13]).

Let δt be the time step size, $t^{n}=n\delta t$, n=0,1,… Let $u^{n}$, $c^{n}$, and $q^{n}$ be the approximations of u, *c*, and *q* at $t^{n}$, respectively. $p^{n-\frac{1}{2}}$ is the approximation of p at $t^{n-\frac{1}{2}}$. Setting $p^{-\frac{1}{2}}=\psi^{-\frac{1}{2}}=0$, in each time interval $\left(t^{n-1},t^{n}\right]$. Therefore, the second-order and fast numerical solution of the chemotaxis-Navier–Stokes equation can be obtained by **Steps 1–5** as follows.

**Step** **1:** The ADI scheme for updating the velocity field:
  (7) $$\left\{\begin{array}{c} \frac{{\tilde{u}}^{n}-u^{n-1}}{\delta t}+\left(u^{*}\cdot \nabla \right)u^{*}+S_{c}\nabla p^{*}-S_{c}∆u^{n-1}=-f\left(q^{*}\right),\\ \frac{{\bar{u}}^{n}-{\tilde{u}}^{n}}{\delta t}-\frac{S_{c}}{2}\partial_{xx}\left({\bar{u}}^{n}-u^{n-1}\right)=0,\\ \frac{u^{n}-{\bar{u}}^{n}}{\delta t}-\frac{S_{c}}{2}\partial_{yy}\left(u^{n}-u^{n-1}\right)=0, \end{array}\right.$$
  where $p^{*}=p^{n-\frac{3}{2}}+\psi^{n-\frac{3}{2}}$, $u^{*}=\frac{3}{2}u^{n-1}-\frac{1}{2}u^{n-2}$, and $q^{*}=\frac{3}{2}q^{n-1}-\frac{1}{2}q^{n-2}$. The variables ${\bar{u}}^{n}$ and $u^{n}$ fulfill the Dirichlet boundary conditions corresponding to the boundaries of the X- and Y-directions, respectively.
**Step** **2:** The dimension splitting scheme for updating the pressure $p^{n-\frac{1}{2}}$:
  (8) $$\left\{\begin{array}{c} {\bar{\psi }}^{n-\frac{1}{2}}-\partial_{xx}{\bar{\psi }}^{n-\frac{1}{2}}=-\frac{\nabla \cdot u^{n}}{\delta t},\\ \psi^{n-\frac{1}{2}}-\partial_{yy}\psi^{n-\frac{1}{2}}={\bar{\psi }}^{n-\frac{1}{2}},\\ p^{n-\frac{1}{2}}=p^{n-\frac{3}{2}}+\psi^{n-\frac{1}{2}}-\frac{1}{2}\chi \eta \nabla \cdot \left(u^{n}+u^{n-1}\right), \end{array}\right.$$
  where 0<χ≤1 is to ensure second-order accuracy. The variables ${\bar{\psi }}^{n-\frac{1}{2}}$ and $\psi^{n-\frac{1}{2}}$ satisfy the homogeneous Neumann boundary conditions corresponding to the boundaries of X- and Y-directions, respectively.
**Step** **3:** The dimension splitting scheme for solving the oxygen concentration *c* based on the first-order operator splitting method:
  (9) $$\left\{\begin{array}{c} \frac{{\bar{c}}^{n}-c^{n-1}}{\delta t}+v_{1}^{n}\partial_{x}c^{n-1}-\zeta \partial_{xx}{\bar{c}}^{n}+\frac{\beta r\left(c^{n-1}\right)q^{n-1}}{2}=0,\\ \frac{c^{n}-{\bar{c}}^{n}}{\delta t}+v_{2}^{n}\partial_{y}{\bar{c}}^{n}-\zeta \partial_{yy}c^{n}+\frac{\beta r\left({\bar{c}}^{n}\right)q^{n-1}}{2}=0, \end{array}\right.$$
  where the variables ${\bar{c}}^{n}$ and $c^{n}$ satisfy the boundary conditions given by (4) corresponding to the X- and Y-directions, respectively.
**Step** **4:** The dimension splitting scheme for solving the cell density *q* based on the first-order operator splitting method:
  (10) $$\left\{\begin{array}{c} \frac{{\bar{q}}^{n}-q^{n-1}}{\delta t}+v_{1}^{n}\partial_{x}q^{n-1}-\partial_{xx}{\bar{q}}^{n}+\alpha \partial_{x}\left(q^{n-1}r\left(c^{n}\right)\partial_{x}c^{n}\right)=0,\\ \frac{q^{n}-{\bar{q}}^{n}}{\delta t}+v_{2}^{n}\partial_{y}{\bar{q}}^{n}-\partial_{yy}q^{n}+\alpha \partial_{y}\left({\bar{q}}^{n}r\left(c^{n}\right)\partial_{y}c^{n}\right)=0. \end{array}\right.$$
  where the variables ${\bar{q}}^{n}$ and $q^{n}$ satisfy the boundary conditions given by (4) corresponding to the X- and Y-directions, respectively.
**Step** **5:** Since the numerical solutions $q^{n}$ and $c^{n}$ have only first-order accuracy in time, this paper uses an extrapolation method to improve the convergence order to second-order. Using **Step 3** and **Step 4**, the time step δt and $\frac{1}{2}\delta t$ are, respectively, used in the time interval $\left[t^{n-1},t^{n}\right]$ to obtain the first-order solutions at $t^{n}$ as $\left(c^{n,1},q^{n,1}\right)$ and $\left(c^{n,2},q^{n,2}\right)$. The numerical solution of u at time $t^{n-\frac{1}{2}}$ is obtained by $\frac{1}{2}\left(u^{n-1}+u^{n}\right)$.

Then, we get second-order solutions by adopting the extrapolation $c^{n}=-c^{n,1}+2c^{n,2}$ and $q^{n}=-q^{n,1}+2q^{n,2}$.

## 3. The MAC Finite Difference Scheme

**Figure 1:**
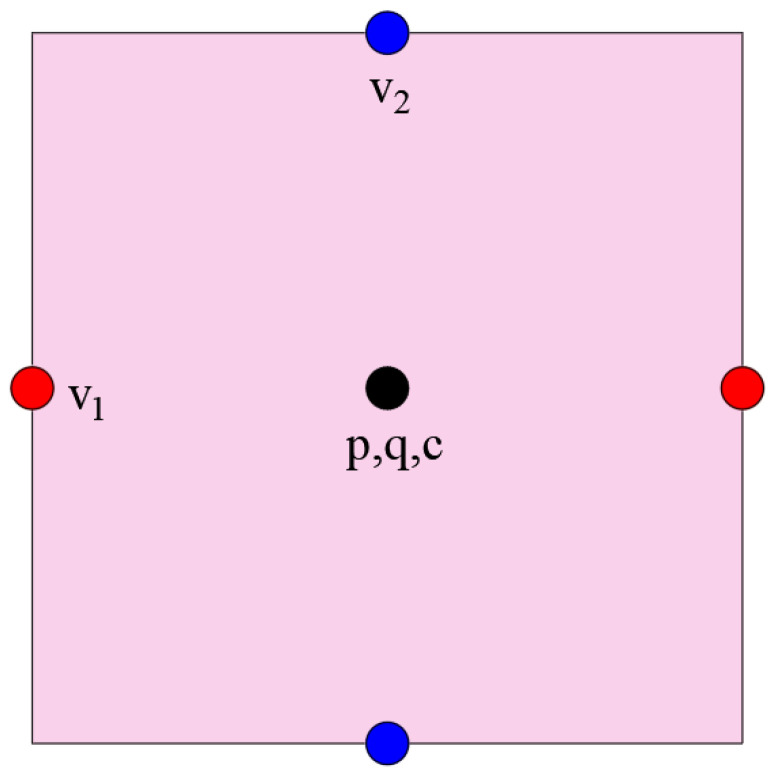
A diagram of the location of variables ($v_{1},v_{2},p,q,c$).

When applying the standard finite difference scheme directly in a standard Cartesian grid for the incompressible Navier–Stokes equation, the resulting numerical pressure is unstable. To address this issue, we use the second-order central difference scheme in the MAC grid configuration to provide a fully discrete scheme for the dimension splitting method above. In the MAC scheme [15], the unknown variable ($v_{1},v_{2},p$) is placed in different positions, as illustrated in Figure 1.

Set the computational domain as $\Omega =\Omega_{x}\times \Omega_{y}=\left[x_{a},x_{b}\right]\times \left[y_{a},y_{b}\right]$, and then a uniform Cartesian grid can be obtained:

(11) $$\begin{array}{ccc} x_{i}=x_{a}+ih_{x}, & i=0,1,2,\ldots ,N_{x}, & h_{x}=\frac{x_{b}-x_{a}}{N_{x}},\\ y_{j}=y_{a}+jh_{y}, & j=0,1,2,\ldots ,N_{y}, & h_{y}=\frac{y_{b}-y_{a}}{N_{y}}, \end{array}$$

where $N_{x}$ and $N_{y}$ are given integers; $x_{a},x_{b}$, and $y_{a},y_{b}$ are the start and end points of Ω in the *x* and *y* directions, respectively. For intermediate nodes, we can define as:

(12) $$\begin{array}{c} {\overset{˘}{x}}_{i}=x_{a}+\overset{˘}{i}h_{x}=x_{a}+\left(i+\frac{1}{2}\right)h_{x},\;i=0,1,2,\ldots ,N_{x}-1,\\ {\overset{˘}{y}}_{j}=y_{a}+\overset{˘}{j}h_{y}=y_{a}+\left(j+\frac{1}{2}\right)h_{y},\;j=0,1,2,\ldots ,N_{y}-1. \end{array}$$

To calculate the gradient of the pressure *p* in the decoupled velocity equation, we define:

(13) $$\begin{array}{c} \;{\hat{\delta }}_{x}p_{i,\overset{˘}{j}}=\frac{p_{\overset{˘}{i},\overset{˘}{j}}-p_{\overset{˘}{i}-1,\overset{˘}{j}}}{h_{x}},\;{\hat{\delta }}_{y}p_{\overset{˘}{i},j}=\frac{p_{\overset{˘}{i},\overset{˘}{j}}-p_{\overset{˘}{i},\overset{˘}{j}-1}}{h_{y}}. \end{array}$$

For the partial derivatives of variables *p*, *q*, and *c* at the cell center, we let U=(p,q,c), $U_{i,j}=U\left(x_{i},y_{j}\right)$, and $U_{\overset{˘}{i},\overset{˘}{j}}=U\left({\overset{˘}{x}}_{i},{\overset{˘}{y}}_{j}\right)$. Define $\nabla_{h}U_{\overset{˘}{i},\overset{˘}{j}}=\left(\delta_{x},\delta_{y}\right)U_{\overset{˘}{i},\overset{˘}{j}}$, and ${∆}_{h}U_{\overset{˘}{i},\overset{˘}{j}}=\left(\delta_{xx}+\delta_{yy}\right)U_{\overset{˘}{i},\overset{˘}{j}}$, where:

(14) $$\begin{array}{c} \delta_{x}U_{\overset{˘}{i},\overset{˘}{j}}=\frac{U_{\overset{˘}{i}+1,\overset{˘}{j}}-U_{\overset{˘}{i}-1,\overset{˘}{j}}}{2h_{x}},\;\delta_{xx}U_{\overset{˘}{i},\overset{˘}{j}}=\frac{U_{\overset{˘}{i}-1,\overset{˘}{j}}-2U_{\overset{˘}{i},\overset{˘}{j}}+U_{\overset{˘}{i}+1,\overset{˘}{j}}}{h_{x}^{2}}, \end{array}$$

(15) $$\begin{array}{c} \delta_{y}U_{\overset{˘}{i},\overset{˘}{j}}=\frac{U_{\overset{˘}{i},\overset{˘}{j}+1}-U_{\overset{˘}{i},\overset{˘}{j}-1}}{2h_{y}},\;\delta_{yy}U_{\overset{˘}{i},\overset{˘}{j}}=\frac{U_{\overset{˘}{i},\overset{˘}{j}-1}-2U_{\overset{˘}{i},\overset{˘}{j}}+U_{\overset{˘}{i},\overset{˘}{j}+1}}{h_{y}^{2}}. \end{array}$$

Define the divergence of velocity on cell center as ${\left({\bar{\nabla }}_{h}\cdot u^{n}\right)}_{\overset{˘}{i},\overset{˘}{j}}={\bar{\delta }}_{x}v_{1,\overset{˘}{i},\overset{˘}{j}}+{\bar{\delta }}_{y}v_{2,\overset{˘}{i},\overset{˘}{j}}$, where:

(16) $$\begin{array}{c} {\bar{\delta }}_{x}v_{1,\overset{˘}{i},\overset{˘}{j}}=\frac{v_{1,i+1,\overset{˘}{j}}-v_{1,i,\overset{˘}{j}}}{h_{x}},\;{\bar{\delta }}_{y}v_{2,\overset{˘}{i},\overset{˘}{j}}=\frac{v_{2,\overset{˘}{i},j+1}-v_{2,\overset{˘}{i},j}}{h_{y}}. \end{array}$$

The first and second partial derivatives of $v_{1}$ at the position of $v_{1}$ are given by:

(17) $$\begin{array}{cc} \delta_{x}^{\left\{1\right\}}v_{1,i,\overset{˘}{j}} & =\frac{v_{1,i+1,\overset{˘}{j}}-v_{1,i-1,\overset{˘}{j}}}{2h_{x}},\;\delta_{xx}^{\left\{1\right\}}v_{1,i,\overset{˘}{j}}=\frac{v_{1,i-1,\overset{˘}{j}}-2v_{1,i,\overset{˘}{j}}+v_{1,i+1,\overset{˘}{j}}}{h_{x}^{2}}, \end{array}$$

(18) $$\begin{array}{cc} \delta_{y}^{\left\{1\right\}}v_{1,i,\overset{˘}{j}} & =\frac{v_{1,i,\overset{˘}{j}+1}-v_{1,i,\overset{˘}{j}-1}}{2h_{y}},\;\delta_{yy}^{\left\{1\right\}}v_{1,i,\overset{˘}{j}}=\frac{v_{1,i,\overset{˘}{j}-1}-2v_{1,i,\overset{˘}{j}}+v_{1,i,\overset{˘}{j}+1}}{h_{y}^{2}}. \end{array}$$

The first and second partial derivatives of $v_{2}$ at the position of $v_{2}$ are given by:

(19) $$\begin{array}{cc} \delta_{x}^{\left\{2\right\}}v_{2,\overset{˘}{i},j} & =\frac{v_{2,\overset{˘}{i}+1,j}-v_{2,\overset{˘}{i}-1,j}}{2h_{x}},\;\delta_{xx}^{\left\{2\right\}}v_{2,\overset{˘}{i},j}=\frac{v_{2,\overset{˘}{i}-1,j}-2v_{2,\overset{˘}{i},j}+v_{2,\overset{˘}{i}+1,j}}{h_{x}^{2}}, \end{array}$$

(20) $$\begin{array}{cc} \delta_{y}^{\left\{2\right\}}v_{2,\overset{˘}{i},j} & =\frac{v_{2,\overset{˘}{i},j+1}-v_{2,\overset{˘}{i},j-1}}{2h_{y}},\;\delta_{yy}^{\left\{2\right\}}v_{2,\overset{˘}{i},j}=\frac{v_{2,\overset{˘}{i},j-1}-2v_{2,\overset{˘}{i},j}+v_{2,\overset{˘}{i},j+1}}{h_{y}^{2}}. \end{array}$$

Then, we let $\nabla_{h}^{\left\{1\right\}}=\left(\delta_{x}^{\left\{1\right\}},\delta_{y}^{\left\{1\right\}}\right)$, $\nabla_{h}^{\left\{2\right\}}=\left(\delta_{x}^{\left\{2\right\}},\delta_{y}^{\left\{2\right\}}\right)$, ${∆}_{h}^{\left\{1\right\}}=\left(\delta_{xx}^{\left\{1\right\}}+\delta_{yy}^{\left\{1\right\}}\right)$, ${∆}_{h}^{\left\{2\right\}}=\left(\delta_{xx}^{\left\{2\right\}}+\delta_{yy}^{\left\{2\right\}}\right)$.

The full discretization for the chemotaxis-Navier–Stokes system can be summarized with the following five steps.

**Step** **1:** $v_{1}^{n}$ can be obtained by sequentially solving three subproblems (21)–(23):
  (21) $$\frac{{\tilde{v}}_{1,i,\overset{˘}{j}}^{n}-v_{1,i,\overset{˘}{j}}^{n-1}}{\delta t}+u_{i,\overset{˘}{j}}\cdot \nabla_{h}^{\left\{1\right\}}v_{1,i,\overset{˘}{j}}+S_{c}\delta_{x}p_{i,\overset{˘}{j}}^{*}-S_{c}{∆}_{h}^{\left\{1\right\}}v_{1,i,\overset{˘}{j}}^{n-1}=-f{\left(q^{*}\right)}_{i,\overset{˘}{j}}.$$
  X-direction: for all $j=0,1,2,\ldots ,N_{y}-1$, solve ${\bar{v}}_{1}^{n}$ by:
  (22) $$\frac{{\bar{v}}_{1,i,\overset{˘}{j}}^{n}-{\tilde{v}}_{1,i,\overset{˘}{j}}^{n}}{\delta t}-\frac{S_{c}}{2}\delta_{xx}^{\left\{1\right\}}\left({\bar{v}}_{1,i,\overset{˘}{j}}^{n}-v_{1,i,\overset{˘}{j}}^{n-1}\right)=0.$$
  Y-direction: for all $i=0,1,2,\ldots ,N_{x}$, solve $v_{1}^{n}$ by:
  (23) $$\frac{v_{1,i,\overset{˘}{j}}^{n}-{\bar{v}}_{1,i,\overset{˘}{j}}^{n}}{\delta t}-\frac{S_{c}}{2}\delta_{yy}^{\left\{1\right\}}\left(v_{1,i,\overset{˘}{j}}^{n}-v_{1,i,\overset{˘}{j}}^{n-1}\right)=0.$$
  $v_{2}^{n}$ can be obtained by sequentially solving three subproblems (24)–(26):
  (24) $$\frac{{\tilde{v}}_{2,\overset{˘}{i},j}^{n}-v_{2,\overset{˘}{i},j}^{n-1}}{\delta t}+u_{\overset{˘}{i},j}\cdot \nabla_{h}^{\left\{2\right\}}v_{2,\overset{˘}{i},j}+S_{c}\delta_{x}p_{\overset{˘}{i},j}^{*}-S_{c}{∆}_{h}^{\left\{2\right\}}v_{2,\overset{˘}{i},j}^{n-1}=-f{\left(q^{*}\right)}_{\overset{˘}{i},j}.$$
  X-direction: for all $j=0,1,2,\ldots ,N_{y}$, solve ${\bar{v}}_{2}^{n}$ by:
  (25) $$\frac{{\bar{v}}_{2,\overset{˘}{i},j}^{n}-{\tilde{v}}_{2,\overset{˘}{i},j}^{n}}{\delta t}-\frac{S_{c}}{2}\delta_{xx}^{\left\{2\right\}}\left({\bar{v}}_{2,\overset{˘}{i},j}^{n}-v_{2,\overset{˘}{i},j}^{n-1}\right)=0.$$
  Y-direction: for all $i=0,1,2,\ldots ,N_{x}-1$, solve $v_{2}^{n}$ by:
  (26) $$\frac{v_{2,\overset{˘}{i},j}^{n}-{\bar{v}}_{2,\overset{˘}{i},j}^{n}}{\delta t}-\frac{S_{c}}{2}\delta_{yy}^{\left\{2\right\}}\left(v_{2,\overset{˘}{i},j}^{n}-v_{2,\overset{˘}{i},j}^{n-1}\right)=0.$$
**Step** **2:** Computing the intermediate variable $\psi^{n}$ by solving X- and Y-direction subproblems and updating pressure:
  X-direction: for all $j=0,1,2,\ldots ,N_{y}-1$, solve ${\bar{\psi }}^{n-\frac{1}{2}}$ by:
  (27) $${\bar{\psi }}_{\overset{˘}{i},\overset{˘}{j}}^{n-\frac{1}{2}}-\delta_{xx}{\bar{\psi }}_{\overset{˘}{i},\overset{˘}{j}}^{n-\frac{1}{2}}=-\frac{{\left({\bar{\nabla }}_{h}\cdot u^{n}\right)}_{\overset{˘}{i},\overset{˘}{j}}}{\delta t}.$$
  Y-direction: for all $i=0,1,2,\ldots ,N_{x}-1$, solve $\psi^{n-\frac{1}{2}}$ by:
  (28) $$\psi_{\overset{˘}{i},\overset{˘}{j}}^{n-\frac{1}{2}}-\delta_{yy}{\hat{\psi }}_{\overset{˘}{i},\overset{˘}{j}}^{n-\frac{1}{2}}=\psi_{\overset{˘}{i},\overset{˘}{j}}^{n-\frac{1}{2}}.$$
  Updating pressure by:
  (29) $$p_{\overset{˘}{i},\overset{˘}{j}}^{n-\frac{1}{2}}=p_{\overset{˘}{i},\overset{˘}{j}}^{n-\frac{3}{2}}+\psi_{\overset{˘}{i},\overset{˘}{j}}^{n-\frac{1}{2}}-\frac{1}{2}\chi \eta {\left({\bar{\nabla }}_{h}\cdot u^{n}+{\bar{\nabla }}_{h}u^{n-1}\right)}_{\overset{˘}{i},\overset{˘}{j}}.$$
**Step** **3:** $c^{n}$ can be obtained by resolving X- and Y-direction subproblems:
  X-direction: for all $j=0,1,2,\ldots ,N_{y}-1$, solve ${\bar{c}}^{n}$ by:
  (30) $$\frac{{\bar{c}}_{\overset{˘}{i},\overset{˘}{j}}^{n}-c_{\overset{˘}{i},\overset{˘}{j}}^{n-1}}{\delta t}+v_{1,\overset{˘}{i},\overset{˘}{j}}^{n}\delta_{x}c_{\overset{˘}{i},\overset{˘}{j}}^{n-1}-\zeta \delta_{xx}{\bar{c}}_{\overset{˘}{i},\overset{˘}{j}}^{n}+\frac{\beta r\left(c_{\overset{˘}{i},\overset{˘}{j}}^{n-1}\right)q_{\overset{˘}{i},\overset{˘}{j}}^{n-1}}{2}=0.$$
  Y-direction: for all $i=0,1,2,\ldots ,N_{x}-1$, solve $c^{n}$ by:
  (31) $$\frac{c_{\overset{˘}{i},\overset{˘}{j}}^{n}-{\bar{c}}_{\overset{˘}{i},\overset{˘}{j}}^{n}}{\delta t}+v_{2,\overset{˘}{i},\overset{˘}{j}}^{n}\delta_{y}{\bar{c}}_{\overset{˘}{i},\overset{˘}{j}}^{n}-\zeta \delta_{yy}c_{\overset{˘}{i},\overset{˘}{j}}^{n}+\frac{\beta r\left({\bar{c}}_{\overset{˘}{i},\overset{˘}{j}}^{n-1}\right)q_{\overset{˘}{i},\overset{˘}{j}}^{n-1}}{2}=0.$$
**Step** **4:** $q^{n}$ can be obtained by resolving X- and Y-direction subproblems:
  X-direction: for all $j=0,1,2,\ldots ,N_{y}-1$, solve ${\bar{q}}^{n}$ by:
  (32) $$\frac{{\bar{q}}_{\overset{˘}{i},\overset{˘}{j}}^{n}-q_{\overset{˘}{i},\overset{˘}{j}}^{n-1}}{\delta t}+v_{1,\overset{˘}{i},\overset{˘}{j}}^{n}\delta_{x}q_{\overset{˘}{i},\overset{˘}{j}}^{n-1}-\delta_{xx}{\bar{q}}_{\overset{˘}{i},\overset{˘}{j}}^{n}+\alpha \delta_{x}\left(q_{\overset{˘}{i},\overset{˘}{j}}^{n-1}r\left(c_{\overset{˘}{i},\overset{˘}{j}}^{n}\right)\delta_{x}c_{\overset{˘}{i},\overset{˘}{j}}^{n}\right)=0.$$
  Y-direction: for all $i=0,1,2,\ldots ,N_{x}-1$, solve $q^{n}$ by:
  (33) $$\frac{q_{\overset{˘}{i},\overset{˘}{j}}^{n}-{\bar{q}}_{\overset{˘}{i},\overset{˘}{j}}^{n}}{\delta t}+v_{2,\overset{˘}{i},\overset{˘}{j}}^{n}\delta_{y}{\bar{q}}_{\overset{˘}{i},\overset{˘}{j}}^{n}-\delta_{yy}q_{\overset{˘}{i},\overset{˘}{j}}^{n}+\alpha \delta_{y}\left({\bar{q}}_{\overset{˘}{i},\overset{˘}{j}}^{n}r\left(c_{\overset{˘}{i},\overset{˘}{j}}^{n}\right)\delta_{y}c_{\overset{˘}{i},\overset{˘}{j}}^{n}\right)=0.$$
  For the discretization of mixed boundary conditions, we achieve this by using the ghost cell technique [4], i.e., the values of virtual points are determined according to the boundary conditions:
  (34) $$q_{i,j+1}:=q_{i,j}e^{\alpha \left(1-c_{i,j}\right)},\;c_{i,j+1}=1,\;q_{i,0}:=q_{i,1},\;c_{i,0}=c_{i,1}.$$

As mentioned above, the two-dimensional chemotaxis-Navier–Stokes model has been decomposed into a series of ODEs and one-dimensional PDEs subproblems, thus requiring only a line-by-line solution. One-dimensional subproblems can be computed in parallel since they can be computed independently of one another in the same direction. Therefore, the dimension splitting method greatly reduces the storage space and improves the computational efficiency.

## 4. Numerical Simulations

### 4.1. Convergence Test

In this part, we test the method’s convergence. The computational domain is set to be $\Omega ={\left[-1,1\right]}^{2}$ for 0≤t≤T:=1. The exact solution for the convergence test is chosen as:

(35) $$\left\{\begin{array}{c} q={\left(x-x^{2}\right)}^{2}{\left(y-y^{2}\right)}^{2}t,\\ c={\left(x-x^{2}\right)}^{2}{\left(y-y^{2}\right)}^{2}t,\\ p=\sin\left(\pi y\right)\cos\left(\pi x\right)\sin\left(t\right),\\ v_{1}=\sin\left(2\pi y\right)\sin^{2}\left(\pi x\right)\pi \sin\left(t\right),\\ v_{2}=-\sin\left(2\pi x\right)\sin^{2}\left(\pi y\right)\pi \sin\left(t\right). \end{array}\right.$$

The parameters α, β, γ, ζ, η, $S_{c}$, and r(c) are set to be one. The value range of χ is 0<χ≤1. However, through our computational experience, most of the models coupled with incompressible NS equations have good numerical performance when $\chi =\frac{1}{2}$. Therefore, this article fixes the χ as $\frac{1}{2}$ based on computational experience. For simplicity, Dirichlet boundary conditions are used for velocity variables and homogeneous Newman boundary conditions for cell and oxygen variables. The spatial discretization size $h_{x}$ and $h_{y}$ is determined by taking {50,75,100,125,150,175,200} divisions in each coordinate direction. To make it easier to verify second-order convergence in space-time, we set the time step size as $\delta t=0.1\min\{h_{x},h_{y}\}$. The error is measured through discrete $l^{2}$-norm, i.e.,

(36) $$l^{2}-\mathrm{error}=\sqrt{\frac{\sum_{i=1}^{N_{x}}\sum_{j=1}^{N_{y}}{\left(U_{i,j}^{n}-U\left(x_{i},y_{j},t^{n}\right)\right)}^{2}}{N_{x}N_{y}}}$$

**Figure 2:**
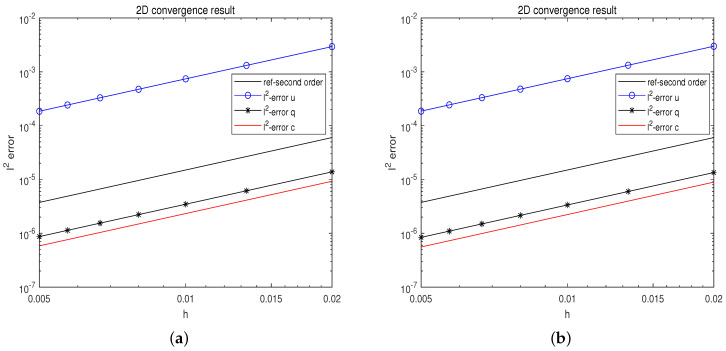
The convergence and accuracy comparison results of the proposed method. (**a**) The proposed dimension splitting method. (**b**) Standard FD discretization without dimension splitting.

Figure 2a displays the $l^{2}$ errors for all unknown variables between the numerical solution and the precise solution at t=1. It is clear that all unknown variables’ error trends are parallel to the second-order reference line, proving that the suggested technique is second-order convergent.

### 4.2. Efficiency Test

All numerical simulations in this article were performed on Matlab2020b, running on an Intel (R) Core (TM) i7-10510u CPU (2.30 GHz) laptop. To evaluate computational efficiency, we compare the time required to solve the chemotaxis-Navier–Stokes equation using dimension splitting and non-dimension splitting schemes. The dimension splitting scheme adopts the spatiotemporal second-order scheme proposed in Section 2 of this paper. The non-dimension splitting scheme uses the second-order projection method in reference [16]. The initial conditions and parameters are the same as in the previous subsection. Table 1 shows the time taken for an iteration. We can see that dimension splitting can significantly improve computational efficiency. For a more rigorous comparison, the computational accuracy of non-dimension splitting and dimension splitting schemes is shown in Figure 2. Their accuracy is comparable and both reach second-order convergence. For storage, in the two-dimensional case, the storage requirement of the non-dimension splitting scheme is $O({\left(N_{x}N_{y}\right)}^{2})$, whereas the dimension splitting method is only $O(N_{x}N_{y})$. To sum up, the dimension splitting scheme proposed in this paper has obvious advantages in efficiency.

### 4.3. Simulating the Biochemotaxis Phenomenon

To numerically simulate the biochemotaxis phenomenon, we ran a number of computational tests on the dimensionless chemotaxis-Navier–Stokes model. Mixed boundary conditions (4) are considered. The step sizes are $h_{x}=0.02$, $h_{y}=0.005$, δt=0.0001. The computational domain is set to be Ω=(3,3)×(0,1).

In all subsequent numerical result graphs shown, the first column is cell density *q*, the second column is chemoattractant concentration *c*, and the third column is the fluid velocity field u. All large values of q≥1 are painted the same shade of red in order to color and emphasize the plumes optimally.

#### 4.3.1. Effect of Increased ζ

In this subsection, we investigate how altering the parameter ζ alters how the cell density changes over time. We perform tests with different values of ζ:ζ=5,25, and 50. Other parameters are fixed as α=10, Sc=500, β=50, η=1, and γ=5000. We set the initial data as:

(37) $$q^{0}\left(x,y\right)=\left\{\begin{array}{c} 1,\;\;\mathrm{if}\;y>0.499-0.01\sin((x-1.5)\pi ),\\ 0.5,\;\;\mathrm{else},\; \end{array}\right.\;c^{0}\left(x,y\right)=1,\;u^{0}\left(x,y\right)=0.$$

**Figure 3:**
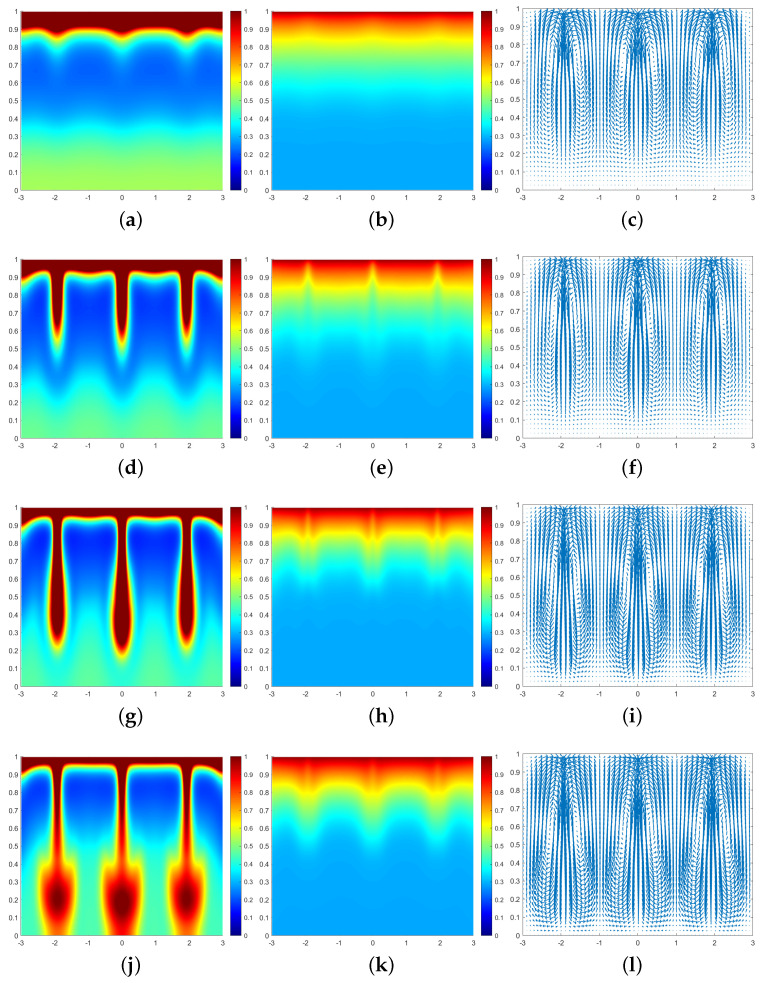
Simulation results of a biochemotaxis phenomenon with ζ=5, β=50, γ=5000. (**a**) *q* at t=0.07. (**b**) *c* at t=0.07. (**c**) u at t=0.07. (**d**) *q* at t=0.092. (**e**) *c* at t=0.092. (**f**) u at t=0.092. (**g**) *q* at t=0.1. (**h**) *c* at t=0.1. (**i**) u at t=0.1. (**j**) *q* at t=0.113. (**k**) *c* at t=0.113. (**l**) u at t=0.113.

**Figure 4:**
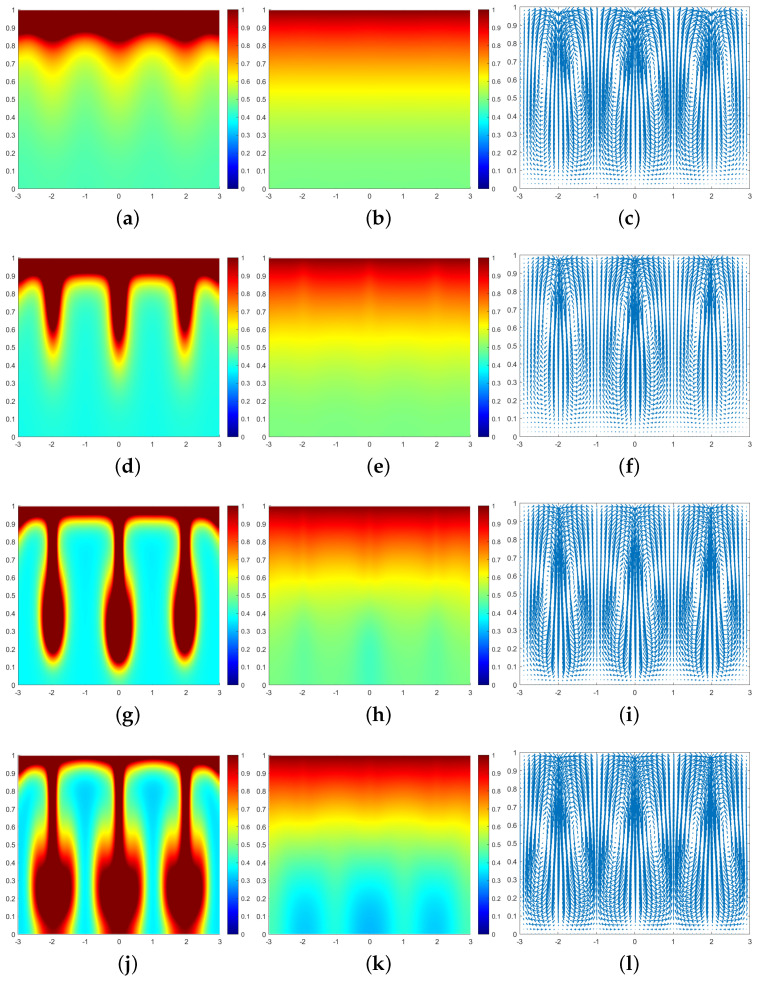
Simulation results of a biochemotaxis phenomenon ζ=25, β=50, γ=5000. (**a**) *q* at t=0.035. (**b**) *c* at t=0.035. (**c**) u at t=0.035. (**d**) *q* at t=0.052. (**e**) *c* at t=0.052. (**f**) u at t=0.052. (**g**) *q* at t=0.06. (**h**) *c* at t=0.06. (**i**) u at t=0.06. (**j**) *q* at t=0.07. (**k**) *c* at t=0.07. (**l**) u at t=0.07.

**Figure 5:**
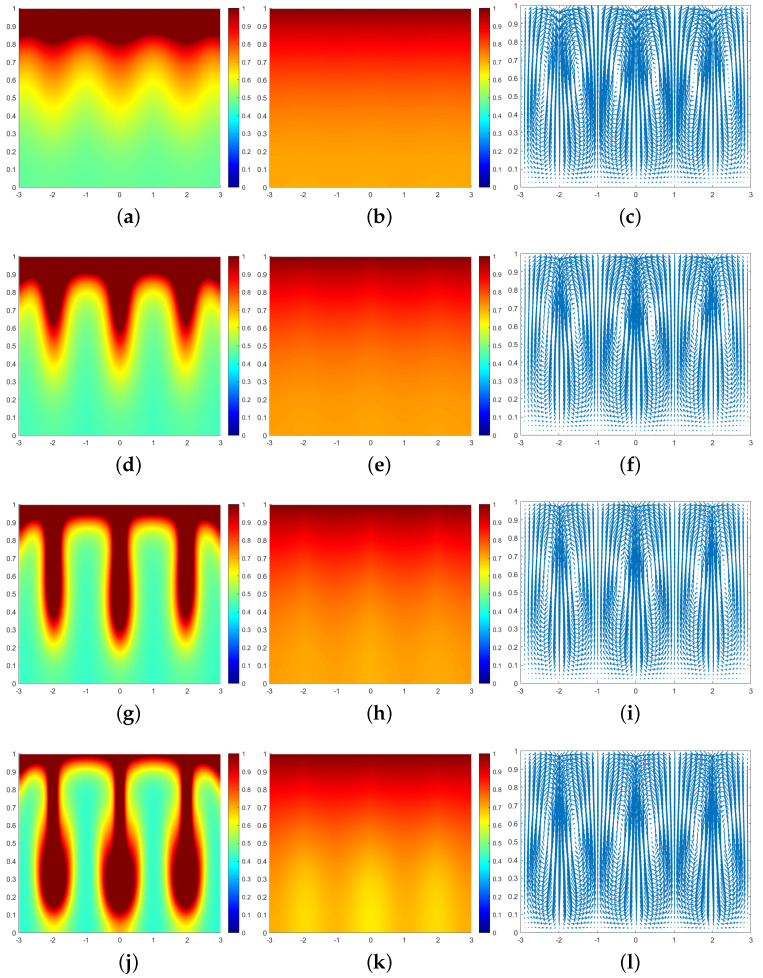
Simulation results of a biochemotaxis phenomenon ζ=50, β=50, γ=5000. (**a**) *q* at t=0.03. (**b**) *c* at t=0.03. (**c**) u at t=0.03. (**d**)*q* at t=0.05. (**e**) *c* at t=0.05. (**f**) u at t=0.05. (**g**) *q* at t=0.058. (**h**) *c* at t=0.058. (**i**) u at t=0.058. (**j**) *q* at t=0.067. (**k**) *c* at t=0.067. (**l**) u at t=0.067.

In Figure 3, we can see that at the moment t=0.07, a large number of cells form a high-concentration cell layer on the air–fluid interface with sufficient oxygen. Due to the Taylor–Rayleigh instability, the biochemotaxis phenomenon occurs and the plume begins to appear. At t=0.092, the plume sinks to the lower half and is finger-shaped. At t=0.1, the lower part of the finger-shaped plume becomes larger. At t=0.113, the finger-shaped plume changes to a mushroom-shaped plume. Upon examining Figure 4 and Figure 5, we can see that the plume appears earlier in each phase. Therefore, we can conclude that as ζ increases, the finger-shaped plume appears earlier and faster, and the shape of the plume changes faster and faster. In other words, increasing the value of ζ accelerates the sinking speed of the plume.

#### 4.3.2. Effect of Increased β and γ

**Figure 6:**
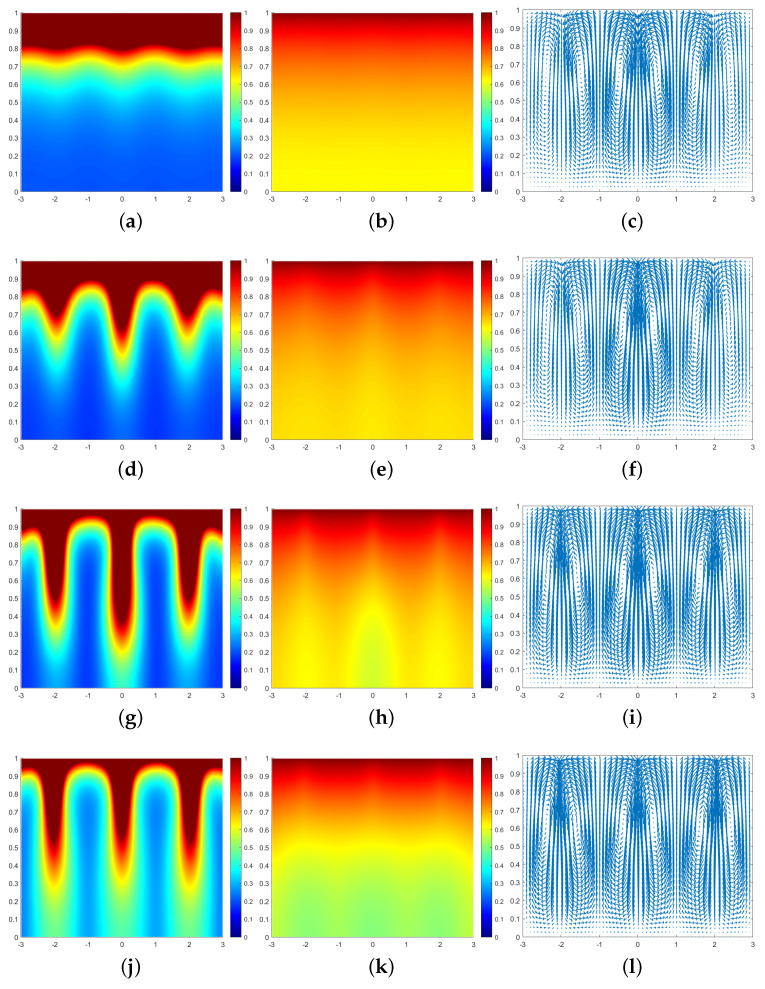
Simulation results of a biochemotaxis phenomenon ζ=5, β=10, γ=1000. (**a**) *q* at t=0.25. (**b**) *c* at t=0.25. (**c**) u at t=0.25. (**d**) *q* at t=0.35. (**e**) *c* at t=0.35. (**f**) u at t=0.35. (**g**) *q* at t=0.4. (**h**) *c* at t=0.4. (**i**) u at t=0.4. (**j**) *q* at t=0.5. (**k**) *c* at t=0.5. (**l**) t=0.5.

**Figure 7:**
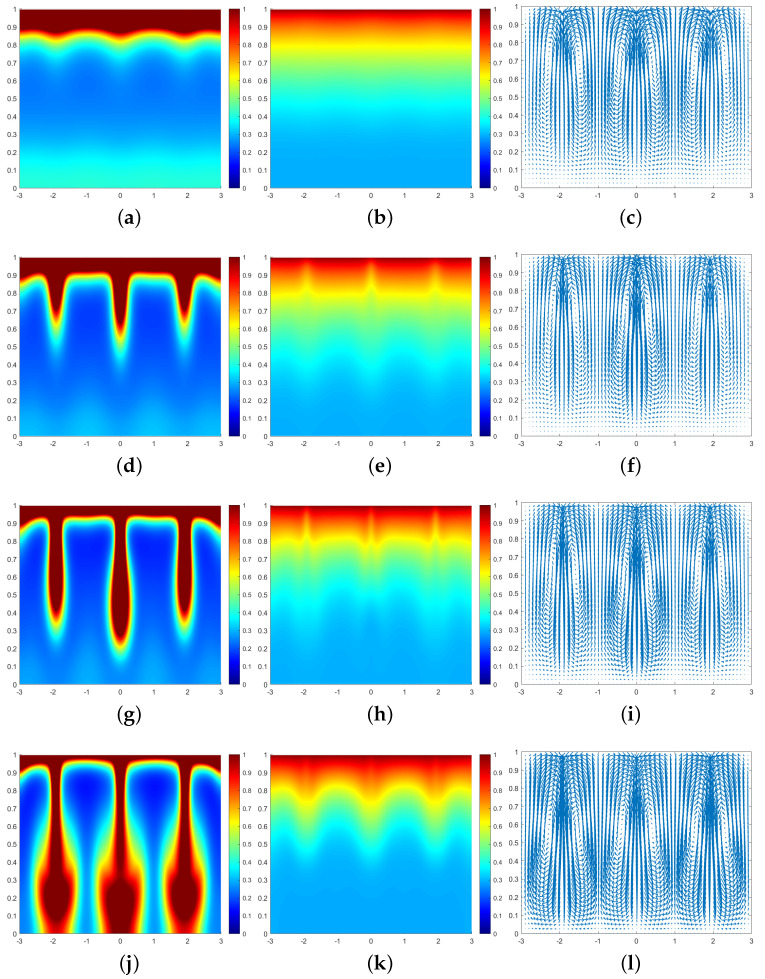
Simulation results of a biochemotaxis phenomenon ζ=5, β=30, γ=3000. (**a**) *q* at t=0.09. (**b**) *c* at t=0.09. (**c**) u at t=0.09. (**d**) *q* at t=0.12. (**e**) *c* at t=0.12. (**f**) u at t=0.12. (**g**) *q* at t=0.13. (**h**) *c* at t=0.13. (**i**) u at t=0.13. (**j**) *q* at t=0.15. (**k**) *c* at t=0.15. (**l**) u at t=0.15.

In this part, we examine what happens to the cell plume as β and γ are increased. Other parameters are fixed as α=10, Sc=500, η=1, and ζ=5. The initial values are still set to (37). Comparing Figure 3, Figure 6 and Figure 7, we can see that the cell plume appears earlier and the plume sinks faster by increasing β and γ.

#### 4.3.3. Test with Random Initial Density of Cells

**Figure 8:**
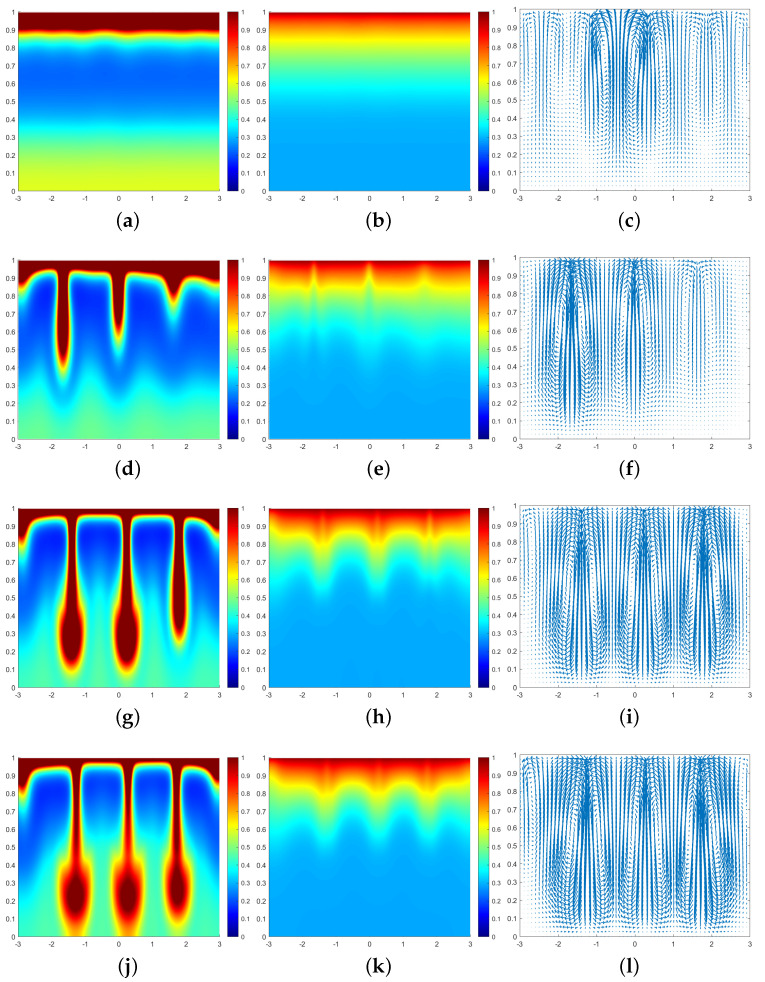
Simulation results of a biochemotaxis phenomenon with initial value $q^{0}\left(x,y\right)=0.8+0.2\cdot rand$. (**a**) *q* at t=0.095. (**b**) *c* at t=0.095. (**c**) u at t=0.095. (**d**) *q* at t=0.13. (**e**) *c* at t=0.13. (**f**) u at t=0.13. (**g**) *q* at t=0.141. (**h**) *c* at t=0.141. (**i**) u at t=0.141. (**j**) *q* at t=0.144. (**k**) *c* at t=0.144. (**l**) u at t=0.144.

The initial density of cells in this paragraph is adjusted to $q^{0}\left(x,y\right)=0.8+0.2\cdot rand$, with rand being a random number uniformly distributed between 0∼1. Other initial values are consistent with (37). The simulation parameters are set as ζ=5, α=10, Sc=500, η=1, β=50, and γ=5000. In Figure 8, we can clearly see that our numerical approach accurately captures the descent dynamics of the plume. The convective instability occurs at t=0.095. Apparently, the plumes are unevenly spaced and sink at different rates. At t=0.141, two rapidly sinking plumes form mushroom shapes. At t=0.144, all the plumes form mushroom shapes.

## 5. Conclusions

A fast dimension splitting scheme for chemotactic-Navier–Stokes systems is proposed. This method combines the pressure correction method with the dimension splitting effect, the ADI method, the operator splitting method, and the extrapolation method to achieve a fast second-order solution. The method decomposes the two-dimensional model into a series of one-dimensional subproblems, thus reducing computational complexity and storage space. The effectiveness and practicability of this method are demonstrated by numerical experiments simulating chemotaxis. Future research will focus on the following directions: (1) the dimension splitting schemes for three-dimensional chemotactic-Navier–Stokes systems: the method proposed in this article can be appropriately modified for solving the coupling system of the incompressible NS equation and the second-order parabolic equation. For example, the effective numerical results of the Allen–Cahn model of two-phase incompressible fluid, the surfactant model of binary fluid, etc., are what we will present in the future. (2) Due to the complexity and nonlinearity of the model, its stability analysis is a challenging problem, which will continue to be solved in the future. (3) The rapid numerical simulation of biochemotaxis in complex regions has more important practical application value. How to establish the dimensional splitting scheme of complex regions is the focus of our follow-up research. 

## Figures and Tables

**Table 1:** Efficiency comparison of the proposed method with non-dimension splitting scheme.

Space Subdivision	*n* = 64	*n* = 128	*n* = 256	*n* = 512	*n* = 1024	*n* = 2048
Dimension splitting	0.194 s	0.154 s	0.260 s	1.543 s	14.745 s	105.419 s
Non-dimension splitting	0.264 s	0.470 s	2.344 s	13.146 s	71.192 s	513.749 s

## Data Availability

No data were used for the research described in the article. The codes generated during the study are all available on request from the corresponding author.
